# Impact of Cardiometabolic Risk Markers on the Incidence and Progression Arterial Stiffness in Patients With Prediabetes

**DOI:** 10.1111/1753-0407.70231

**Published:** 2026-05-08

**Authors:** Qi Sun, Qiujing Cai, Yilin Huang, Shuyu Wang, Lisheng Liu, Zhiguang Liu, Aihua Hu, Wei Li, Biyan Wang

**Affiliations:** ^1^ Precision and Intelligence Medical Imaging Lab, Beijing Friendship Hospital Capital Medical University Beijing China; ^2^ Fuwai Hospital, Chinese Academy of Medical Sciences & Peking Union Medical College/National Center for Cardiovascular Diseases Beijing China; ^3^ Beijing Hypertension League Beijing China; ^4^ Clinical Trial Unit, Beijing Anzhen Hospital Capital Medical University Beijing China; ^5^ Center for Non‐Communicable Disease Management, Beijing Children's Hospital Capital Medical University, National Center for Children's Health Beijing China

**Keywords:** arterial stiffness, cardiometabolic risk markers, insulin resistance, mean arterial pressure, prediabetes

## Abstract

**Background:**

Previous studies have reported that insulin resistance (IR), blood pressure, body mass index (BMI), and dyslipidemia are associated with arterial stiffness. However, limited evidence exists regarding these relationships in prediabetes patients. The purpose of this study was to investigate the longitudinal associations between cardiometabolic risk markers and progression of arterial stiffness.

**Methods:**

This study included 5771 adults with prediabetes at baseline from the Shougang cohort study. Cardiometabolic risk markers included the triglyceride glucose (TyG) index, triglyceride to high‐density lipoprotein cholesterol (TG/HDL‐C) ratio, mean arterial pressure (MAP), BMI, and dyslipidemia. Arterial stiffness was assessed using brachial‐ankle pulse wave velocity (baPWV), and its progression was defined as the annual change in baPWV between baseline and the last visit. Multivariate linear and logistic regression models were used to investigate the associations.

**Results:**

Multivariate linear regression analyses showed TyG index, TG/HDL‐C ratio, MAP, and dyslipidemia were significantly associated with baseline baPWV. Over a mean follow‐up of 3.24 years, each one‐unit increase in the MAP was significantly associated with a faster progression of baPWV (difference, 0.72 cm/s per year; 95% CI, 0.40 to 1.04 cm/s per year). After adjusting for all covariates, the risk for arterial stiffness increased 3% with each one‐unit increase of MAP.

**Conclusion:**

Among individuals with prediabetes, MAP showed consistent longitudinal associations with arterial stiffness, highlighting the importance of early blood pressure management for vascular protection prior to the onset of diabetes.

## Highlights

- Mean arterial pressure was linked to both progression and new onset of arterial stiffness among prediabetes patients.
- Blood pressure control plays a more direct role than other metabolic factors in preventing arterial stiffening.

## Graphical abstract

Early blood pressure management may offer a more effective strategy to prevent vascular complications in this population.

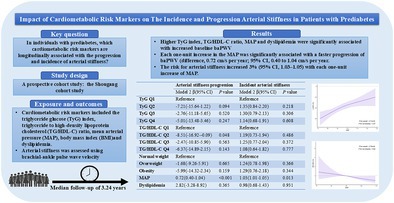

## 1 Introduction

Patients with prediabetes (preD) are at high risk of progressing to overt diabetes and cardiovascular disease (CVD), which has imposed a heavy economic burden globally. In 2045, 10.0% of adults around the world were estimated to have preD [1]. Arterial stiffness is the earliest functional damage in the process of vascular aging, and is a well‐established predictor of incident CVD and mortality [2, 3, 4]. Brachia‐ankle pulse wave velocity (baPWV) measurement is widely used in clinical setting to evaluate arterial stiffness. Prior to diabetes, the base of macrovascular complication already acted in the prediabetes phase; prediabetes is associated with more advanced vascular damage compared with normal populations [5, 6, 7]. Therefore, given that patients with preD are at elevated cardiovascular risk, it is crucial to clarify the influence of cardiometabolic risk markers on arterial stiffness in this population.

There are several underlying different pathways of the link between preD and vascular damage; one of the major mechanisms is metabolic syndrome, such as insulin resistance (IR), obesity, and dyslipidemia [8, 9, 10]. Triglyceride‐glucose (TyG) index and the triglycerides to high‐density lipoprotein (TG/HDL) ratio were considered a reliable and inexpensive surrogate biomarker of IR [11, 12]. Some previous studies have been conducted to indicate a positive association between IR [13, 14, 15], obesity [16, 17], dyslipidemia, and blood pressure [18]. However, those studies are all focused on general populations. Recently, Maurizio et al. found that the TG/HDL, rather than TyG, was independently associated with baPWV in preD patients. However, this study is a cross‐sectional study without follow‐up and is limited by relatively small sample sizes [19]. In addition, no study has comprehensively evaluated the association between cardiometabolic risk factors and arterial stiffness progression in preD patients. In the present study, we extend previous findings and provide a longitudinal perspective analysis to explore the associations between cardiometabolic risk markers (TyG index, TG/HDL‐C, body mass index [BMI], mean arterial blood pressure [MAP], and dyslipidemia) and the progression and incidence of arterial stiffness measured by baPWV among preD populations.

## 2 Methods

### 2.1 Study Population and Design

The Shougang cohort study in north China is a prospective community‐based cohort established between 2011 and 2012 in Beijing, China, with new individuals recruited biennially. This longitudinal cohort study was designed to assess the determinants and progression of CVD. The study based on health examination populations from the community in north China, and the recruited individuals were asked to take a biennially health examination, including demographic features and lifestyle factors assessed by face‐to‐face questionnaire survey, anthropometric measurements, and blood tests. The study design and procedures were detailed previously [20, 21].

**FIGURE 1:**
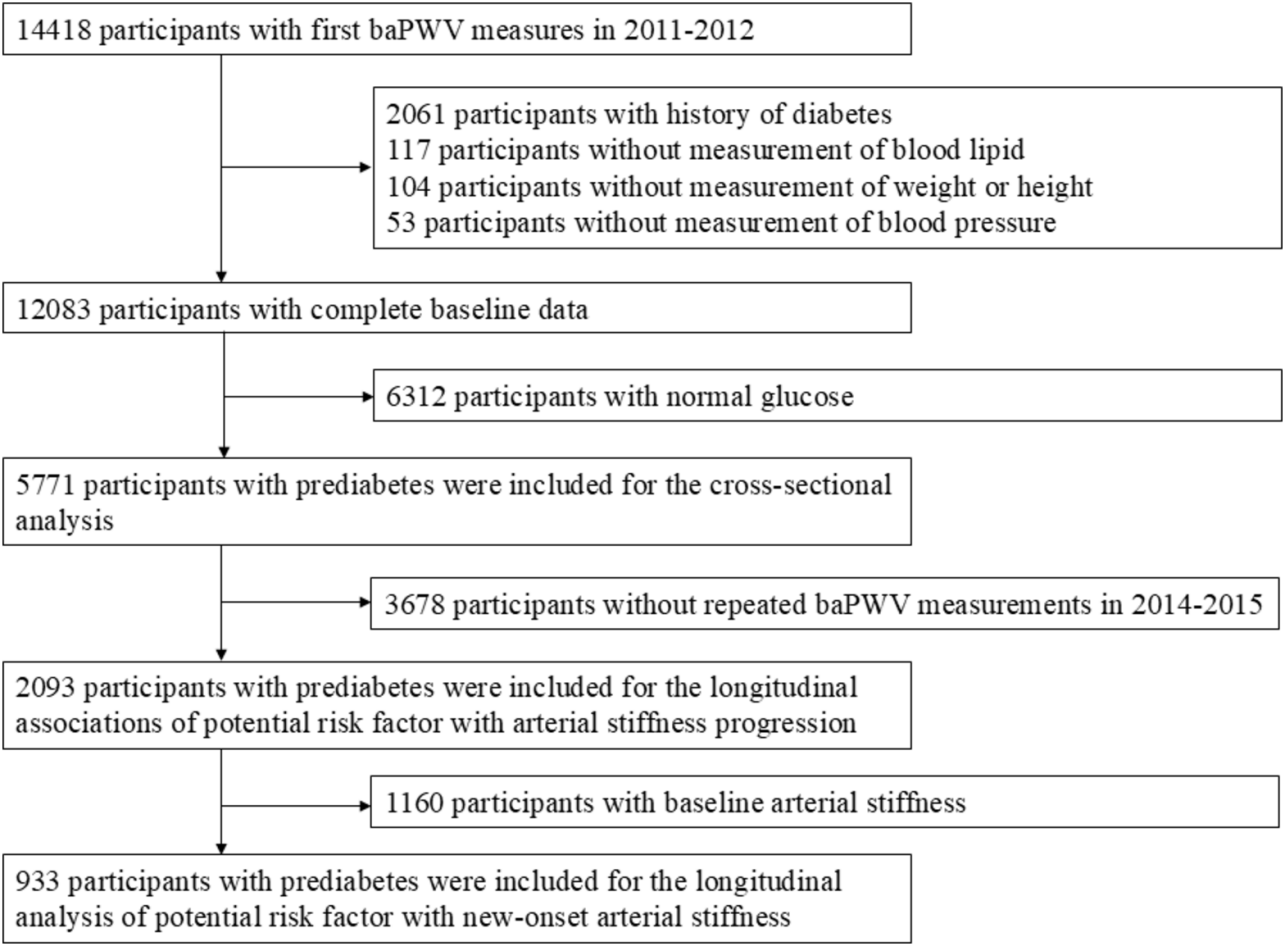
Flow chart of the study population.

Initially, 14 418 individuals who underwent baPWV examination at baseline were recruited in this current study. Of those participants, 2061 had a confirmed history of diabetes. Additionally, 117 participants without lipid measurements, 104 without weight or height data, and 53 without blood pressure measurements were excluded. For the cross‐sectional analysis of cardiometabolic risk markers (TyG index, TG/HDL‐C, BMI, MAP, and dyslipidemia) and baseline baPWV, 5771 participants were included. Furthermore, for the analysis of the association between cardiometabolic risk factors and arterial stiffness progression, we excluded 3708 participants without repeated baPWV measurements, leaving 2063 individuals. Additionally, for the analysis of arterial stiffness incidence, we included 933 participants without arterial stiffness at baseline (defined as baPWV < 1400 cm/s). The detailed flowchart is shown in Figure 1. The survey protocols, instruments, and informed consent procedures were approved by the Ethics Committee of Beijing Hypertension League Institute (Ethical Approval No. 2017‐102), and all participants provided written informed consent. This study was conducted in accordance with the principles of the Declaration of Helsinki.

### 2.2 Definition of Prediabetes

Prediabetes was identified by fasting blood glucose (FBG) level between 5.6 and 6.9 mmol/L, or a 2‐h plasma glucose (PG) level between 7.8 and 11.0 mmol/L during a 75 g oral glucose tolerance test (OGTT) [22]. FBG was defined as blood glucose levels measured after an overnight fast of 8–10 h, during which only water was allowed, while two‐hour postprandial PG was measured 2 h after the start of a standardized meal, using capillary blood obtained via fingerstick.

### 2.3 BaPWV Measurement and Definition of Arterial Stiffness Progression and Incidence

BaPWV was measured using the BP‐203 RPE III networked AS detection device (Omron Health Medical Co. Ltd., China), as detailed elsewhere [21]. After not smoking and resting for at least 5 min, participants were instructed to lie in a supine position and remain quiet during the measurement. Pressure waveforms from the brachial and post tibial were recorded by cuffs placed around all arms and ankles. The device measured the time differences between the initial rise of pulse waveforms at the brachial and tibial sites during systole. Based on participants' height, the distance between the two points was automatically estimated. The baPWV was then calculated by dividing the estimated distance by the measured time interval. The measurement of baPWV was undertaken by trained nurses at baseline and follow‐up. For this analysis, the average value of the left and right baPWV was used.

Arterial stiffness progression was calculated using the value of baPWV change between baseline and last visit, divided by the follow‐up time in years [23]. Arterial stiffness incidence was defined using a cut‐off point of baPWV ≥ 1400 cm/s during follow‐up among participants without arterial stiffness at baseline [24].

### 2.4 Definition of Cardiometabolic Risk Markers

Weight and height were measured using standardized protocols by trained staff. Body mass index (BMI) was calculated as weight/height^2^ (kg/m^2^). We then categorized the participants as normal weight (18.5–23.9 kg/m^2^), overweight (24–27.9 kg/m^2^), or obesity (≥ 28 kg/m^2^) according to the recommendations in China [25, 26]. Systolic blood pressures (SBP) and diastolic blood pressures (DBP) were measured in the seated position three times by one trained examiner using an automatic blood pressure monitor (Omron HEM 7200 Monitor), and the average of three readings was calculated. Mean arterial blood pressure (MAP) was calculated as 1/3*SBP + 2/3*DBP [27].

The blood samples were transported and stored at −80°C refrigerator of the Peking University Shougang Hospital in Beijing. Triglycerides (TG), low‐density lipoprotein cholesterol (LDL‐C), high‐density lipoprotein cholesterol (HDL‐C), and total cholesterol (TC) were acquired following a standard protocol of biochemical detection. Dyslipidemia was defined as TG ≥ 2.3 mmol/L, TC ≥ 6.2 mmol/L, LDL‐C ≥ 4.1 mmol/L, or HDL‐C < 1.0 mmol/L according to the Guidelines on Prevention and Treatment of Dyslipidemia [28]. The TyG index was defined as ln [TG (mm/L) * FBG (mm/L)/2]. The TG/HDL‐C ratio was calculated as TG (mm/L)/HDL‐C (mm/L).

### 2.5 Data Collection and Assessment of Covariates

The demographic characteristics (e.g., age, sex, marital status, educational levels), lifestyle (smoking status, drinking status, physical activity, sleep duration), history of diseases and medication information were collected via a standard questionnaire. Marital status was categorized into two groups: currently married versus non‐married. Education was classified as low (primary school or below), intermediate (junior to senior high school), and high (college or above). Smoking and drinking status were classed into: current and former or never. Regular physical activity was defined as ≥ 80 min/week. Adequate sleep duration was defined as 6–9 h/day, including insomnia and nighttime.

### 2.6 Statistical Analysis

The baseline characteristics were described as mean and standard deviation for continuous variables and frequency for categorical variables. Differences across prevalence, incidence of arterial stiffness and baPWV change were compared using chi‐square test, ANOVA, or Kruskal–Wallis H test, respectively. Because missing data were minimal (never exceeding 0.3%), the single‐imputation method was performed for missing values.

Initially, we explored the cross‐sectional associations of cardiometabolic risk markers (TyG index, TG/HDL‐C ratio, BMI, MAP, and dyslipidemia) with baseline baPWV using multivariate linear regression. To adjust for potential confounding factors, two models were established as follows: model 1 adjusted for age and sex; model 2 adjusted for age, sex, marital status, educational levels, smoking status, drinking status, physical activity, sleep duration, lipid‐lowering, antihypertensive medications, and history of coronary heart disease or stroke. In the analysis of the progression of baPWV, baPWV at baseline was additionally adjusted in the linear regression models together with baseline baPWV. Furthermore, multivariable‐adjusted logistic regression models were used to estimate the odds ratios (ORs) and the corresponding 95% confidence interval (CI) for the associations between cardiometabolic risk markers and the risk of incident arterial stiffness during the follow‐up among participants who were free of arterial stiffness at baseline after adjusting for the aforementioned covariates. Additionally, we performed restricted cubic spline analysis with three knots positioned at the 10th, 50th, and 90th percentiles to examine the dose–response relationship between MAP and both the progression and risk of arterial stiffness.

Several sensitivity analyses were also performed to assess the robustness of the results. First, multivariable‐adjusted logistic regression models were conducted to assess the association of cardiometabolic risk markers with prevalence of arterial stiffness, which was defined as baseline baPWV ≥ 1400 cm/s. Second, both the TyG index and TG/HDL‐C ratio were analyzed as continuous variables and as categorical variables based on quartile cut‐offs. Third, we excluded participants who were undergoing treatment with lipid‐lowering and antihypertensive medications. Fourth, based on follow‐up glycemic status, participants with prediabetes were classified into three groups: regression to euglycemia (normal FBG and 2‐h postprandial PG), persistent prediabetes, and progression to diabetes, we further examined the associations between cardiometabolic risk factors and baPWV progression among the three groups.

All statistical analyses were performed with R statistical software (version 3.6.3), and SAS (version 9.4). A two‐sided *p* < 0.05 was considered as the threshold for statistical significance.

## 3 Results

### 3.1 Baseline Characteristics of the Study Population

The cross‐sectional analysis included 5771 preD individuals. The mean age was 57.50 ± 8.81 years, and 2069 (35.85%) were men. The median baseline baPWV was 1581 cm/s. Meanwhile, among 2093 preD participants included in the longitudinal analysis, the median baseline age was 57.35 ± 8.41 years, and 738 (35.26%) were men. The median values of the absolute baPWV change, the annual baPWV change rate, and the baPWV slope were 46 cm/s, 9 cm/s/year and 8.11, respectively, as shown in Table 1.

**TABLE 1:** Characteristics of the study population.

	Participants of corss‐sectional analysis	Participants of longitudinal analysis
No. of participants	5771	2093
Age (years)	57.70 (8.81)	57.35 (8.41)
Total cholesterol (mmol/L)	5.40 (1.02)	5.42 (1.06)
Triglyceride (mmol/L)	1.70 (1.22)	1.71 (1.26)
SBP (mmHg)	135.38 (16.61)	135.06 (16.40)
DBP (mmHg)	75.74 (9.74)	75.83 (9.59)
baPWV at baseline (cm/s)	1581 (1399, 1830.50)	1563.50 (1389, 1816)
BMI	26.46 (3.36)	26.44 (3.32)
TyG index	8.83 (0.55)	8.84 (0.55)
HDL‐C (mmol/L)	1.41 (0.36)	1.41 (0.36)
LDL‐C (mmol/L)	3.33 (0.83)	3.34 (0.83)
MAP (mmHg)	95.62 (10.59)	95.58 (10.51)
TG/HDL ratio	1.40 (1.51)	1.42 (1.55)
Sex (*n*, %)		
Male	2069 (35.85)	738 (35.26)
Female	3702 (64.15)	1355 (64.74)
Current married (*n*, %)	5365 (92.96)	1951 (93.22)
Educational level (*n*, %)		
Primary school or below	479 (8.30)	143 (6.83)
Junior to senior high school	4221 (73.14)	1559 (74.49)
College or above	1071 (18.56)	391 (18.68)
Lipid‐lowering medication (*n*, %)	385 (6.67)	7 (0.33)
Antihypertensive medication (*n*, %)	1504 (26.06)	233 (11.13)
History of CHD (*n*, %)	502 (8.70)	143 (6.83)
History of stroke (*n*, %)	185 (3.21)	52 (2.48)
Adequate sleep duration (*n*, %)	3381 (58.59)	1452 (69.37)
Current smoker (*n*, %)	1259 (21.82)	372 (17.77)
Current drinking (n, %)	1572 (27.24)	659 (31.49)
Regular physical activity (*n*, %)	4148 (71.88)	1405 (67.13)
Normal weight	1300 (22.53)	471 (22.50)
Overweight	2731 (47.32)	998 (47.68)
Obesity	1740 (30.15)	624 (29.81)
Dyslipidaemia (*n*, %)	2219 (38.45)	814 (38.89)
Progression of baPWV (cm/s/years)	—	−39.67 (−96.27, 2.65)

*Note:* Data are the mean (SD), median [IQR] or number (%).

Abbreviations: baPWV, brachial‐ankle pulse wave velocity; BMI, body mass index; CHD, coronary heart disease; DBP, diastolic blood pressure; HDL‐C, high‐density lipoprotein cholesterol; LDL‐C, low‐density lipoprotein cholesterol; MAP, mean arterial pressure; SBP, systolic blood pressure; TyG, triglyceride glucose.

### 3.2 Association Between Cardiometabolic Risk Markers and Baseline baPWV in Cross‐Sectional Study

The cross‐sectional associations between cardiometabolic risk markers and baseline baPWV among preD participants are shown in Table 2. Overall, there were significant positive associations of TyG index, TG/HDL‐C ratio, MAP, and dyslipidemia with baPWV at baseline. In the multivariate‐adjusted linear regression models, the adjusted *β* (95% CIs) in the second, third, and highest quartiles of TyG index were 26.01 (4.10–47.92), 46.75 (24.81–68.68), and 95.24 (73.20–117.27), respectively, when the lowest quartile as a reference. For TG/HDL‐C ratio, the adjusted β for participants were 27.18 (5.26–49.10), 32.71 (10.75–54.67), 73.99 (51.84–96.15). Similarly, each unit increase in TyG and TG/HDL‐C was associated with a 69.63 and 14.72 cm/s increase in baPWV, respectively. Additionally, each unit increase in MAP was associated with a 12.34 (11.64–13.03) cm/s higher baseline baPWV. PreD individuals with dyslipidemia had significantly higher baPWV than those without, with a difference of 29.59 (13.59–45.59) cm/s.

**TABLE 2:** Association of cardiometabolic risk markers with baPWV at baseline in linear models (*N* = 5771).

	Model 1 *β* (95% CI)	*p*	Model 2 *β* (95% CI)	*p*
TyG Q1	Reference		Reference	
TyG Q2	28.49 (6.42–50.55)	0.011	26.01 (4.10–47.92)	0.020
TyG Q3	49.95 (27.89–72.01)	< 0.001	46.75 (24.81–68.68)	< 0.001
TyG Q4	99.02 (76.98–121.05)	< 0.001	95.24 (73.20–117.27)	< 0.001
TyG index (continuous)	71.55 (57.30–85.81)	< 0.001	69.63 (55.36–83.90)	< 0.001
TG/HDL‐C Q1	Reference		Reference	
TG/HDL‐C Q2	31.30 (9.20–53.40)	0.006	27.18 (5.26–49.10)	0.015
TG/HDL‐C Q3	35.07 (12.94–57.19)	0.002	32.71 (10.75–54.67)	0.004
TG/HDL‐C Q4	79.02 (56.84–101.21)	< 0.001	73.99 (51.84–96.15)	< 0.001
TG/HDL‐C ratio (continuous)	15.27 (10.06–20.49)	< 0.001	14.72 (9.53–19.91)	< 0.001
Normal weight	Reference		Reference	
Overweight	17.72 (−2.39–37.84)	0.084	11.83 (−8.15 to 31.80)	0.246
Obesity	31.56 (9.70–53.43)	0.005	16.26 (−5.71 to 38.23)	0.147
MAP	12.63 (11.95–13.31)	< 0.001	12.34 (11.64–13.03)	< 0.001
Dyslipidemia	30.92 (14.80–47.04)	< 0.001	29.59 (13.59–45.59)	< 0.001

*Note:* Model 1, adjusted for age and sex at baseline. Model 2, adjusted for variables in model 1 plus marital status, educational levels, smoking status, drinking status, physical activity, sleep duration, lipid‐lowering, antihypertensive medications, and history of coronary heart disease or stroke at baseline.

Abbreviations: baPWV, brachial‐ankle pulse wave velocity; CI, confidence interval; HDL‐C, high‐density lipoprotein cholesterol; MAP, mean arterial pressure; Q, quartiles; TG, triglyceride; TyG index, triglyceride–glucose index.

### 3.3 Associations of Cardiometabolic Risk Factors With the Arterial Stiffness Progression in the Longitudinal Study Population

We further investigated the longitudinal associations of cardiometabolic risk factors with the progression of baPWV, and found that the growth rate of baPWV had a statistically significant linear increment in participants with a high level of MAP (Table 3 and Figure S1). A multivariable linear regression model showed that a one‐unit increase in the MAP was significantly associated with a faster progression of baPWV (difference, 0.72 cm/s/year; 95% CI, 0.40 to 1.04 cm/s/year), after adjusting for baseline baPWV and other risk factors. While other cardiometabolic risk factors did not show significant associations with baPWV progression. In sensitivity analyses stratified by glycemic status during follow‐up, the associations between cardiometabolic markers and baPWV progression remained largely consistent with the main findings, which MAP showed a robust association with baPWV progression particularly among persistent prediabetes (Table S1).

**TABLE 3:** Association of cardiometabolic risk markers with baPWV progression in follow‐ups (*N* = 2093).

	Model 1 *β* (95% CI)	*p*	Model 2 *β* (95% CI)	*p*
TyG Q1	Reference		Reference	
TyG Q2	−7.95 (−16.37 to 0.47)	0.064	−7.21 (−15.64 to1.22)	0.094
TyG Q3	−5.75 (−14.15 to 2.64)	0.179	−2.76 (−11.18 to 5.65)	0.520
TyG Q4	−3.27 (−11.67 to 5.12)	0.445	−5.01 (−13.48 to 3.46)	0.247
TyG index (continuous)	−2.14 (−7.53 to 3.25)	0.437	−1.46 (−6.91 to 3.99)	0.600
TG/HDL‐C Q1	Reference		Reference	
TG/HDL‐C Q2	−8.71 (−17.13 to 0.30)	0.043	−8.51 (−16.92 to 0.09)	0.048
TG/HDL‐C Q3	−7.02 (−15.48 to 1.44)	0.104	−2.47 (−10.85 to 5.90)	0.563
TG/HDL‐C Q4	−3.45 (−11.82 to 4.92)	0.419	−6.37 (−14.89 to 2.15)	0.143
TG/HDL‐C ratio (continuous)	−0.72 (−2.65 to 1.21)	0.467	−0.51 (−2.45 to 1.43)	0.606
Normal weight	Reference		Reference	
Overweight	−1.49 (−9.07 to 6.09)	0.700	−1.68 (−9.26 to 5.91)	0.665
Obesity	−5.89 (−14.17 to 2.38)	0.163	−5.99 (−14.32 to 2.34)	0.159
MAP	0.76 (0.44–1.08)	< 0.001	0.72 (0.40–1.04)	< 0.001
Dyslipidemia	3.03 (−3.05 to 9.10)	0.329	2.82 (−3.28 to 8.92)	0.365

*Note:* Model 1, adjusted for age and sex at baseline. Model 2, adjusted for variables in model 1 plus baPWV at baseline, marital status, educational levels, smoking status, drinking status, physical activity, sleep duration, lipid‐lowering, antihypertensive medications, and history of coronary heart disease or stroke at baseline.

Abbreviations: baPWV, brachial‐ankle pulse wave velocity; CI, confidence interval; HDL‐C, high‐density lipoprotein cholesterol; MAP, mean arterial pressure; Q, quartiles; TG, triglyceride; TyG index, triglyceride–glucose index.

### 3.4 Associations of Cardiometabolic Risk Factors With Arterial Stiffness Incidence in the Longitudinal Study Population

During a mean follow‐up of 3.24 (2.38–3.33) years, there were 164 incident cases of arterial stiffness among the 933 participants. After adjusting for all covariates, the risk for arterial stiffness increased 3% (95% CI, 1.03–1.05, *p* = 0.013) with each one‐unit increase of MAP, with no evidence of non‐linearity (Table 4 and Figure S2). In sensitivity analysis, the results did not show material changes for cardiometabolic risk factors and the prevalence of arterial stiffness defined by baseline baPWV ≥ 1400 cm/s (Table S2), or excluding participants with lipid‐lowering or antihypertensive medications (Tables S3–S5).

**TABLE 4:** Association of cardiometabolic risk markers with incident arterial stiffness in follow‐ups (*N* = 933).

	Model 1 OR (95% CI)	*p*	Model 2 OR (95% CI)	*p*
TyG Q1	Reference		Reference	
TyG Q2	1.24 (0.77–1.98)	0.378	1.35 (0.84–2.20)	0.218
TyG Q3	1.23 (0.76–2.00)	0.402	1.30 (0.79–2.13)	0.306
TyG Q4	1.08 (0.66–1.78)	0.762	1.14 (0.68–1.91)	0.608
TyG index (continuous)	1.14 (0.83–1.56)	0.417	1.17 (0.84–1.62)	0.354
TG/HDL‐C Q1	Reference		Reference	
TG/HDL‐C Q2	1.17 (0.73–1.90)	0.511	1.19 (0.73–1.94)	0.486
TG/HDL‐C Q3	1.24 (0.77–2.01)	0.372	1.25 (0.77–2.04)	0.372
TG/HDL‐C Q4	1.05 (0.64–1.75)	0.836	1.08 (0.64–1.82)	0.777
TG/HDL‐C ratio (continuous)	0.99 (0.88–1.11)	0.833	0.98 (0.87–1.11)	0.763
Normal weight	Reference		Reference	
Overweight	1.21 (0.76–1.91)	0.423	1.24 (0.78–1.98)	0.366
Obesity	1.35 (0.81–2.24)	0.25	1.29 (0.76–2.18)	0.344
MAP	1.03 (1.01–1.06)	0.003	1.03 (1.01–1.05)	0.013
Dyslipidemia	1.08 (0.75–1.55)	0.684	0.98 (0.68–1.43)	0.931

*Note:* Model 1, adjusted for age and sex at baseline. Model 2, adjusted for variables in model 1 plus marital status, educational levels, smoking status, drinking status, physical activity, sleep duration, lipid‐lowering, antihypertensive medications, and history of coronary heart disease or stroke at baseline.

Abbreviations: baPWV, brachial‐ankle pulse wave velocity; CI, confidence interval; HDL‐C, high‐density lipoprotein cholesterol; MAP, mean arterial pressure; OR, odds ratio; Q, quartiles; TG, triglyceride; TyG index, triglyceride–glucose index.

## 4 Discussion

In this study of individuals with prediabetes, we evaluated multiple cardiometabolic risk markers in relation to arterial stiffness. We identified a significant association of the MAP with baPWV as a quantitative marker of arterial stiffness among preD patients. The MAP was related to baPWV at baseline as well as the annual growth rate of baPWV. Furthermore, the MAP was observed to be associated with the incidence of arterial stiffness. To the best of our knowledge, this is the first study to reveal the relationship between the cardiometabolic risk markers (TyG index, TG/HDL‐C, BMI, MAP, and dyslipidemia) and the progression of arterial stiffness in a prospective cohort. These findings highlight the differentiated predictive value of hemodynamic versus metabolic indicators in early vascular changes among individuals with prediabetes.

Our study found that MAP was the only cardiometabolic risk marker significantly associated with both the arterial stiffness progression and incidence in prediabetes populations. After adjusting for a wide range of confounders, each unit increase in MAP was associated with a measurable increase in baPWV progression rate and a 3% higher risk of incident arterial stiffness. The association between MAP and the annualized rate of baPWV increase suggests that even in prediabetes, chronic hemodynamic stress is increasingly recognized as a significant factor contributing to subclinical vascular deterioration. That indicated in the context of prediabetes, where glucose metabolism is impaired but not yet at the level of diabetes, the role of MAP becomes even more crucial. This finding is supported by previous studies showing that MAP reflects sustained pressure load on the arterial wall, promoting structural remodeling such as collagen deposition, medial thickening, and elastic fiber degradation [29]. Unlike systolic or diastolic pressure alone, MAP captures cumulative vascular stress during the entire cardiac cycle [30]. What's more, this supports previous findings that sustained pressure load can promote arterial wall remodeling [31, 32, 33], including vascular smooth muscle hypertrophy and increased collagen deposition. In the context of prediabetes, where metabolic changes may be intermittent or reversible, MAP appears to exert a more consistent mechanical burden on arteries, accelerating functional and structural vascular change. Given that MAP is routinely measured and modifiable, it may serve as a practical and early intervention target in individuals with prediabetes to prevent downstream cardiovascular damage. Our findings underscore the value of MAP not only as a BP metric, but also as an early biomarker for cumulative vascular burden in early disease stages.

We observed that the TyG index and TG/HDL‐C ratio were positively associated with baPWV at baseline, but neither was predictive of arterial stiffness progression nor incident during follow‐up. These indices, widely used as markers of insulin resistance, reflect early‐stage metabolic stress through dysregulated glucose and lipid metabolism [13, 34]. Mechanistically, insulin resistance contributes to endothelial dysfunction by reducing nitric oxide availability, increasing oxidative stress, and triggering low‐grade inflammation [35, 36, 37, 38]. These pathways can increase arterial stiffness in the short term, primarily through functional impairment of vascular tone and compliance. However, they may not be sufficient to drive the long‐term structural changes required for sustained baPWV elevation. Additionally, both TyG index and TG/HDL‐C are dynamic indices sensitive to lifestyle interventions. In prediabetic individuals, metabolic improvement during follow‐up (such as weight loss, improved glycemic control) may weaken their long‐term vascular impact. This could partly explain the lack of longitudinal associations, highlighting a limitation of using static metabolic markers to predict progressive vascular damage.

Higher BMI is often linked to increased sympathetic tone, systemic inflammation, and vascular stiffness [39, 40], while dyslipidemia contributes to endothelial dysfunction and oxidative stress [41, 42]. However, in our study, neither BMI nor dyslipidemia had an association with arterial stiffness progression or incidence, possibly due to limited follow‐up time, measurement at a single timepoint, or their indirect effects being mediated through other factors such as BP. These findings suggest that while BMI and lipid status reflect vascular health at a given point, they may not serve as reliable markers for predicting future arterial deterioration in prediabetes.

Our results underscore the importance of recognizing heterogeneity within the prediabetic population. While insulin resistance may initiate vascular dysfunction, not all individuals with elevated TyG or TG/HDL‐C experience progressive arterial damage. This reinforces the concept that blood pressure burden, rather than short‐term metabolic disturbance, plays a dominant role in vascular aging in this group. It also aligns with previous findings suggesting that prediabetes is a transitional, and potentially reversible, metabolic stage [43]. The observed association between MAP and arterial stiffness progression was confined to individuals with sustained prediabetes, but not those who reverted to normoglycemia or progressed to diabetes. This suggests that persistent metabolic dysregulation, in the absence of clinical intervention, may render the vasculature more susceptible to pressure‐induced damage. Thus, while metabolic abnormalities should still be addressed for overall cardiometabolic health, blood pressure, specifically MAP, may be a more actionable and stable target for preventing vascular complications in prediabetic populations.

From a practical standpoint, both MAP and baPWV are non‐invasive, cost‐effective, and feasible for implementation in workplace or community health settings. Integrating these two measurements could support early identification of individuals with prediabetes who are at high risk for vascular damage, enabling targeted lifestyle or pharmacologic interventions. This has broader public health significance, especially in China and other countries where prediabetes prevalence is rising among younger populations. Through blood pressure control before diabetes develops may offer a critical window of opportunity to prevent long‐term cardiovascular disease.

There are several potential limitations in our study. We only assessed TyG and TG/HDL‐C at baseline, without accounting for their temporal changes. This may have underestimated their long‐term effects. In addition, BMI and lipid parameters were measured only once, which may underestimate their cumulative effects. Additionally, as an observational study, causality cannot be definitively inferred. Future work should explore dynamic metabolic trajectories and their interaction with blood pressure and arterial aging. Finally, the relatively short follow‐up duration may not fully capture the long‐term effects of all cardiometabolic risk markers on arterial stiffness. Longer follow‐up period studies are warranted to confirm these findings.

## 5 Conclusions

In the prediabetic population, MAP was the only cardiometabolic marker associated with both the progression and new onset of arterial stiffness. Although TyG index, TG/HDL‐C ratio and dyslipidemia were linked to higher baseline baPWV, they did not predict long‐term vascular changes. These findings emphasize the importance of routine blood pressure monitoring, especially MAP, for early vascular risk identification. While managing traditional cardiometabolic risk factors remains essential, blood pressure control may play a more direct role in preventing arterial stiffening in individuals with prediabetes.

## Author Contributions

**Qi Sun:** writing – review and editing, funding acquisition. **Qiujing Cai:** conceptualization, methodology, software, writing – original draft. **Yilin Huang:** conceptualization, methodology software, writing – original draft. **Shuyu Wang:** data curation, investigation. **Lisheng Liu:** data curation, investigation. **Zhiguang Liu:** methodology, software. **Aihua Hu:** writing – review and editing, funding acquisition. **Wei Li:** writing – review and editing, funding acquisition. **Biyan Wang:** conceptualization, methodology, software, writing – original draft.

## Funding

This work was supported by the National Natural Science Foundation of China (Grant No.: 52500256), the National Center for Cardiovascular Diseases, the National Clinical Research Center for Cardiovascular Diseases, Fuwai Hospital, Shenzhen High‐level Hospital Construction Fund (Grant No.: NCRCSZ‐2023‐012), and the National Center for Health Services Research and Development (Grant No.: WKZX2022JG051).

## Ethics Statement

The survey protocols, instruments, and informed consent procedures were approved by the Ethics Committee of Beijing Hypertension League Institute (Ethical Approval No. 2017‐102), and all participants provided written informed consent.

## Consent

The authors confirm that patient consent forms have been obtained for this article.

## Conflicts of Interest

The authors declare no conflicts of interest.

## Supporting information


**Table S1:** Association of cardiometabolic risk markers with baPWV progression in follow‐ups classified based on follow‐up glycemic status.
**Table S2:** Association of cardiometabolic risk markers with prevalence of arterial stiffness (defined as baseline baPWV ≥ 1400 cm/s).
**Table S3:** Association of cardiometabolic risk markers with baPWV at baseline in linear models after excluded participants with lipid‐lowering or antihypertensive medications (*N* = 4141).
**Table S4:** Association of cardiometabolic risk markers with baPWV progression in follow‐ups after excluded participants with lipid‐lowering or antihypertensive medications (*N* = 1855).
**Table S5:** Association of cardiometabolic risk markers with incident arterial stiffness in follow‐ups after excluded participants with lipid‐lowering or antihypertensive medications (*N* = 826).
**Figure S1:** The associations of mean arterial blood pressure with the progression of arterial stiffness.
**Figure S2:** The associations of mean arterial blood pressure with the risk of arterial stiffness.

## Data Availability

The data that support the findings of this study are available from the corresponding author upon reasonable request.
