# Supplementary material for: Impact of Cardiometabolic Risk Markers on the Incidence and Progression Arterial Stiffness in Patients With Prediabetes
Source: J Diabetes. 2026 May 8;18(5):e70231. doi: 10.1111/1753-0407.70231 (PMC13156238; doi:10.1111/1753-0407.70231)
Supplement: Supplementary file 1 — Table S1: Association of cardiometabolic risk markers with baPWV progression in follow‐ups classified based on follow‐up glycemic status. Table S2: Association of cardiometabolic risk markers with prevalence of arterial stiffness (defined as baseline baPWV ≥ 1400 cm/s). Table S3: Association of cardiometabolic risk markers with baPWV at baseline in linear models after excluded participants with lipid‐lowering or antihypertensive medications (N = 4141). Table S4: Association of cardiometabolic risk markers with baPWV progression in follow‐ups after excluded participants with lipid‐lowering or antihypertensive medications (N = 1855). Table S5: Association of cardiometabolic risk markers with incident arterial stiffness in follow‐ups after excluded participants with lipid‐lowering or antihypertensive medications (N = 826). Figure S1: The associations of mean arterial blood pressure with the progression of arterial stiffness. Figure S2: The associations of mean arterial blood pressure with the risk of arterial stiffness. [file JDB-18-e70231-s001.docx]

**Online-Only Supplemental Material**

**Impact of Cardiometabolic Risk Markers on The Incidence and Progression Arterial Stiffness in Patients with Prediabetes**

Supplement table 1. Association of cardiometabolic risk markers with baPWV progression in follow-ups classified based on follow-up glycemic status.

Supplement table 2. Association of cardiometabolic risk markers with prevalence of arterial stiffness (defined as baseline baPWV ≥ 1400 cm/s).

Supplement table 3. Association of cardiometabolic risk markers with baPWV at baseline in linear models after excluded participants with lipid-lowering or antihypertensive medications (N=4141).

Supplement table 4. Association of cardiometabolic risk markers with baPWV progression in follow-ups after excluded participants with lipid-lowering or antihypertensive medications (N=1855).

Supplement table 5. Association of cardiometabolic risk markers with incident arterial stiffness in follow-ups after excluded participants with lipid-lowering or antihypertensive medications (N=826).

Supplement Figure 1. The associations of mean arterial blood pressure with the progression of arterial stiffness.

Supplement Figure 2. The associations of mean arterial blood pressure with the risk of arterial stiffness.

**Supplement table 1. Association of cardiometabolic risk markers with baPWV progression in follow-ups classified based on follow-up glycemic status.**

|  | **Regression to euglycemia (N=559)** | | | | **Persistent prediabetes(N=1074)** | | | | **Progression to diabetes(N=430)** | | | |  |
| --- | --- | --- | --- | --- | --- | --- | --- | --- | --- | --- | --- | --- | --- |
|  | **Model 1 β(95% CI)** | ***P*** | **Model 2 β(95% CI)** | ***P*** | **Model 1 β(95% CI)** | ***P*** | **Model 2 β(95% CI)** | ***P*** | **Model 1 β(95% CI)** | ***P*** | **Model 2 β(95% CI)** | ***P*** |  |
| TyG |  |  |  |  |  |  |  |  |  |  |  |  |  |
| Q1 | Reference |  | Reference |  |  |  |  |  |  |  |  |  |  |
| Q2 | 1.57(-11.50-14.64) | 0.814 | 1.18(-12.12-14.48) | 0.862 | -9.20(-21.81-3.41) | 0.153 | -8.42(-21.07-4.23) | 0.193 | -23.05(-44.07--2.03) | 0.032 | -20.98(-42.01-0.05) | 0.051 |  |
| Q3 | -5.65(-19.61-8.31) | 0.428 | -6.33(-20.42-7.76) | 0.379 | -4.59(-16.97-7.80) | 0.468 | -3.88(-16.30-8.55) | 0.541 | -7.14(-28.08-13.80) | 0.504 | -5.73(-26.78-15.32) | 0.594 |  |
| Q4 | -1.23(-14.77-12.32) | 0.859 | -1.50(-15.20-12.20) | 0.830 | -6.01(-18.84-6.83) | 0.359 | -4.55(-17.54-8.44) | 0.492 | -16.91(-36.51-2.69) | 0.092 | -12.77(-32.55-7.01) | 0.206 |  |
| TG/HDL-C |  |  |  |  |  |  |  |  |  |  |  |  |  |
| Q1 | Reference |  | Reference |  |  |  |  |  |  |  |  |  |  |
| Q2 | -10.71(-24.10-2.68) | 0.118 | -11.08(-24.62-2.47) | 0.110 | -9.78(-22.23-2.67) | 0.124 | -9.57(-22.01-2.87) | 0.132 | -6.78(-27.71-14.15) | 0.526 | -7.54(-28.53-13.45) | 0.482 |  |
| Q3 | -4.86(-18.43-8.71) | 0.483 | -5.65(-19.38-8.09) | 0.421 | -3.81(-16.44-8.83) | 0.555 | -2.65(-15.25-9.95) | 0.680 | -3.77(-23.33-15.80) | 0.706 | -0.05(-19.64-19.55) | 0.996 |  |
| Q4 | -7.95(-21.69-5.80) | 0.258 | -8.52(-22.40-5.36) | 0.229 | -9.91(-22.72-2.92) | 0.130 | -9.01(-21.99-3.98) | 0.174 | -0.80(-20.29-18.69) | 0.936 | 1.13(-18.32-20.58) | 0.910 |  |
| Normal weight | Reference |  | Reference |  |  |  |  |  |  |  |  |  |  |
| Overweight | 4.44(-7.16-16.05) | 0.453 | 4.43(-7.27-16.12) | 0.459 | -7.13(-18.42-4.16) | 0.216 | -7.06(-18.37-4.24) | 0.221 | 1.41(-18.37-21.19) | 0.889 | 1.30(-18.59-21.19) | 0.898 |  |
| Obesity | -10.52(-24.11-3.07) | 0.130 | -11.18(-24.90-2.53) | 0.111 | -9.07(-21.32-3.18) | 0.147 | -8.09(-20.45-4.27) | 0.200 | 0.41(-20.16-20.99) | 0.969 | -1.57(-22.28-19.14) | 0.882 |  |
| MAP | 0.31(-0.23-0.85) | 0.265 | 0.28(-0.27-0.83) | 0.321 | 1.01(0.53-1.49) | <0.001 | 0.96(0.48-1.44) | <0.001 | 0.59(-0.09-1.26) | 0.091 | 0.50(-0.18-1.19) | 0.149 |  |
| Dyslipidemia | -3.07(-13.34-7.19) | 0.558 | -2.97(-13.33-7.40) | 0.575 | 5.71(-3.31-14.72) | 0.215 | 4.99(-4.11-14.09) | 0.283 | 4.58(-8.92-18.07) | 0.507 | 2.41(-11.18-15.99) | 0.728 |  |

TyG index, triglyceride–glucose index; baPWV, brachial-ankle pulse wave velocity; CI, confidence interval; Q, quartiles; MAP, mean arterial pressure; BMI, body mass index; HDL-C, high-density lipoprotein cholesterol; TG, triglyceride.

Model 1, adjusted for age and sex at baseline.

Model 2, adjusted for variables in model 1 plus marital status, educational levels, smoking status, drinking status, physical activity, sleep duration, lipid-lowering, antihypertensive medications, and history of coronary heart disease or stroke at baseline.

**Supplement table 2. Association of cardiometabolic risk markers with prevalence of arterial stiffness (defined as baseline baPWV ≥ 1400 cm/s).**

|  | **Model 1 β(95% CI)** | ***P* value** | **Model 2 β(95% CI)** | ***P* value** |
| --- | --- | --- | --- | --- |
| TyG Q1 | Reference |  | Reference |  |
| TyG Q2 | 1.37(1.14-1.63) | <0.001 | 1.34(1.12-1.61) | 0.001 |
| TyG Q3 | 1.71(1.42-2.05) | <0.001 | 1.67(1.39-2.01) | <0.001 |
| TyG Q4 | 1.94(1.61-2.33) | <0.001 | 1.90(1.58-2.29) | <0.001 |
| TyG index (continuous) | 1.63(1.44-1.84) | <0.001 | 1.61(1.42-1.82) | <0.001 |
| TG/HDL-C Q1 | Reference |  | Reference |  |
| TG/HDL-C Q2 | 1.27(1.06-1.51) | 0.009 | 1.23(1.03-1.48) | 0.022 |
| TG/HDL-C Q3 | 1.57(1.31-1.89) | <0.001 | 1.55(1.29-1.86) | <0.001 |
| TG/HDL-C Q4 | 1.82(1.51-2.18) | <0.001 | 1.77(1.46-2.13) | <0.001 |
| TG/HDL-C ratio (continuous) | 1.11(1.05-1.17) | <0.001 | 1.10(1.05-1.16) | 0.001 |
| Normal weight | Reference |  | Reference |  |
| Overweight | 1.15(0.97-1.35) | 0.101 | 1.09(0.93-1.29) | 0.295 |
| Obesity | 1.49(1.24-1.78) | <0.001 | 1.32(1.10-1.59) | 0.003 |
| MAP | 1.11(1.10-1.12) | <0.001 | 1.10(1.09-1.11) | <0.001 |
| Dyslipidemia | 0.90(0.79-1.03) | 0.123 | 0.91(0.80-1.04) | 0.178 |

TyG index, triglyceride–glucose index; baPWV, brachial-ankle pulse wave velocity; CI, confidence interval; Q, quartiles; MAP, mean arterial pressure; HDL-C, high-density lipoprotein cholesterol; TG, triglyceride.

Model 1, adjusted for age and sex at baseline.

Model 2, adjusted for variables in model 1 plus marital status, educational levels, smoking status, drinking status, physical activity, sleep duration, lipid-lowering, antihypertensive medications, and history of coronary heart disease or stroke at baseline.

**Supplement table 3. Association of cardiometabolic risk markers with baPWV at baseline in linear models after excluded participants with lipid-lowering or antihypertensive medications (N=4141).**

|  | **Model 1 β(95% CI)** | ***P* value** | **Model 2 β(95% CI)** | ***P* value** |
| --- | --- | --- | --- | --- |
| TyG Q1 | Reference |  | Reference |  |
| TyG Q2 | 33.716(9.352-58.081) | 0.007 | 34.395(10.019-58.771) | 0.006 |
| TyG Q3 | 52.808(28.148-77.469) | <0.001 | 54.767(30.087-79.447) | <0.001 |
| TyG Q4 | 100.313(75.573-125.053) | <0.001 | 100.758(75.890-125.626) | <0.001 |
| TG/HDL-C Q1 | Reference |  | Reference |  |
| TG/HDL-C Q2 | 23.792(-0.773-48.357) | 0.058 | 25.232(0.663-49.800) | 0.044 |
| TG/HDL-C Q3 | 38.745(14.161-63.329) | 0.002 | 38.341(13.762-62.920) | 0.002 |
| TG/HDL-C Q4 | 74.301(49.257-99.344) | <0.001 | 74.193(49.063-99.323) | <0.001 |
| Normal weight | Reference |  | Reference |  |
| Overweight | 23.383(1.466-45.299) | 0.037 | 21.295(-0.621-43.210) | 0.057 |
| Obesity | 37.020(12.342-61.699) | 0.003 | 33.023(8.261-57.785) | 0.009 |
| MAP | 12.646(11.861-13.431) | <0.001 | 12.656(11.872-13.439) | <0.001 |
| Dyslipidemia | 28.833(10.545-47.120) | 0.002 | 28.935(10.635-47.236) | 0.002 |

TyG index, triglyceride–glucose index; baPWV, brachial-ankle pulse wave velocity; CI, confidence interval; Q, quartiles; MAP, mean arterial pressure; HDL-C, high-density lipoprotein cholesterol; TG, triglyceride.

Model 1, adjusted for age and sex at baseline.

Model 2, adjusted for variables in model 1 plus marital status, educational levels, smoking status, drinking status, physical activity, sleep duration, lipid-lowering, antihypertensive medications, and history of coronary heart disease or stroke at baseline.

**Supplement table 4. Association of cardiometabolic risk markers with baPWV progression in follow-ups after excluded participants with lipid-lowering or antihypertensive medications (N=1855).**

|  | **Model 1 β(95% CI)** | ***P*** | **Model 2 β(95% CI)** | ***P*** |
| --- | --- | --- | --- | --- |
| TyG Q1 | Reference |  | Reference |  |
| TyG Q2 | -7.24(-16.01-1.53) | 0.1059 | -6.52(-15.31-2.27) | 0.1464 |
| TyG Q3 | -4.55(-13.36-4.27) | 0.312 | -4.28(-13.13-4.56) | 0.3423 |
| TyG Q4 | -3.78(-12.61-5.04) | 0.4009 | -3.09(-11.98-5.81) | 0.4969 |
| TG/HDL-C Q1 | Reference |  | Reference |  |
| TG/HDL-C Q2 | -9.05(-17.87--0.23) | 0.0445 | -8.94(-17.77--0.11) | 0.0474 |
| TG/HDL-C Q3 | -6.76(-15.73-2.21) | 0.1396 | -4.50(-13.23-4.24) | 0.3133 |
| TG/HDL-C Q4 | -5.10(-13.82-3.61) | 0.2514 | -5.89(-14.91-3.13) | 0.2004 |
| Normal weight | Reference |  | Reference |  |
| Overweight | -1.16(-9.02-6.69) | 0.7718 | -0.86(-8.74-7.02) | 0.8304 |
| Obesity | -8.43(-17.12-0.26) | 0.0575 | -7.60(-16.33-1.13) | 0.0881 |
| MAP | 0.63(0.29-0.97) | <0.001 | 0.60(0.26-0.94) | 0.0006 |
| Dyslipidemia | 1.60(-4.82-8.01) | 0.6262 | 1.36(-5.08-7.80) | 0.6782 |

TyG index, triglyceride–glucose index; baPWV, brachial-ankle pulse wave velocity; CI, confidence interval; Q, quartiles; MAP, mean arterial pressure; HDL-C, high-density lipoprotein cholesterol; TG, triglyceride.

Model 1, adjusted for age and sex at baseline.

Model 2, adjusted for variables in model 1 plus baPWV at baseline, marital status, educational levels, smoking status, drinking status, physical activity, sleep duration, lipid-lowering, antihypertensive medications, and history of coronary heart disease or stroke at baseline.

**Supplement table 5. Association of cardiometabolic risk markers with incident arterial stiffness in follow-ups after excluded participants with lipid-lowering or antihypertensive medications (N=826).**

|  | **Model 1 β(95% CI)** | ***P*** | **Model 2 β(95% CI)** | ***P*** |
| --- | --- | --- | --- | --- |
| TyG Q1 | Reference |  | Reference |  |
| TyG Q2 | 1.38(0.82-2.30) | 0.224 | 1.48(0.87-2.50) | 0.149 |
| TyG Q3 | 1.37(0.79-2.35) | 0.262 | 1.51(0.86-2.65) | 0.15 |
| TyG Q4 | 1.21(0.69-2.12) | 0.504 | 1.36(0.76-2.42) | 0.303 |
| TG/HDL-C Q1 | Reference |  | Reference |  |
| TG/HDL-C Q2 | 1.36(0.80-2.30) | 0.254 | 1.36(0.79-2.32) | 0.264 |
| TG/HDL-C Q3 | 1.42(0.83-2.41) | 0.198 | 1.40(0.81-2.41) | 0.224 |
| TG/HDL-C Q4 | 1.16(0.65-2.05) | 0.617 | 1.23(0.68-2.23) | 0.493 |
| Normal weight | Reference |  | Reference |  |
| Overweight | 1.10(0.67-1.80) | 0.703 | 1.14(0.69-1.87) | 0.617 |
| Obesity | 1.26(0.73-2.19) | 0.410 | 1.26(0.72-2.21) | 0.425 |
| MAP | 1.04(1.02-1.07) | 0.002 | 1.03(1.01-1.07) | 0.003 |
| Dyslipidemia | 0.99(0.66-1.47) | 0.942 | 0.91(0.60-1.37) | 0.641 |

TyG index, triglyceride–glucose index; baPWV, brachial-ankle pulse wave velocity; CI, confidence interval; Q, quartiles; MAP, mean arterial pressure; HDL-C, high-density lipoprotein cholesterol; TG, triglyceride.

Model 1, adjusted for age and sex at baseline.

Model 2, adjusted for variables in model 1 plus marital status, educational levels, smoking status, drinking status, physical activity, sleep duration, lipid-lowering, antihypertensive medications, and history of coronary heart disease or stroke at baseline.

**Supplement Figure 1. The associations of mean arterial blood pressure with the progression of arterial stiffness.**


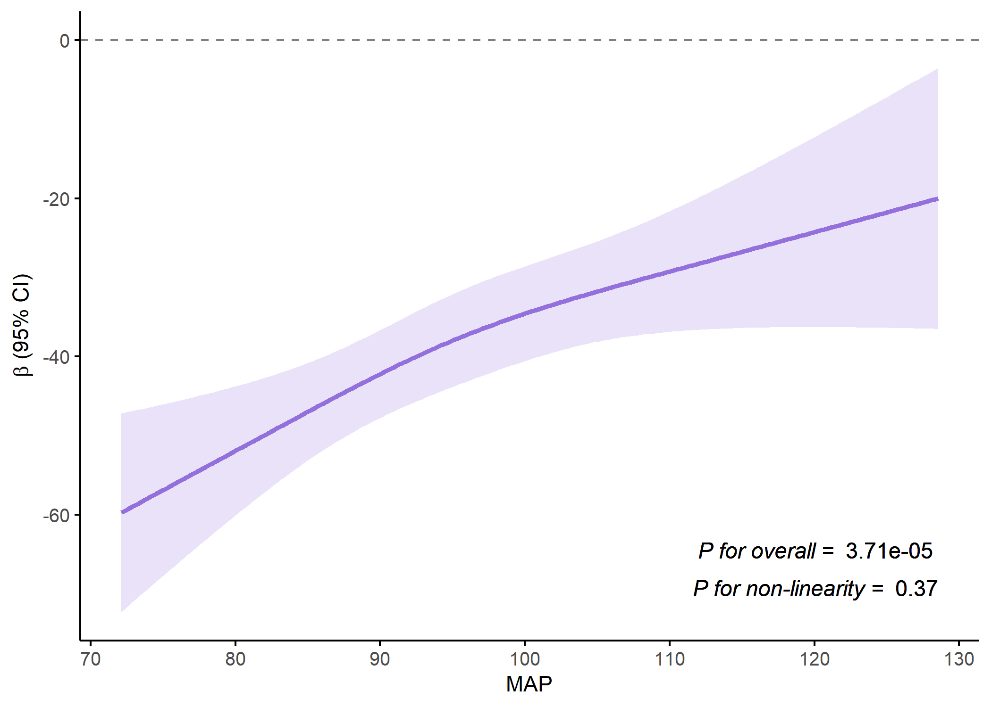


The data were fitted using restricted cubic spline linear regression models with three knots placed at the 10th, 50th, and 90th percentiles of baseline mean arterial pressure (MAP).

MAP: mean arterial blood pressure, CI: confidence interval.

Covariates in the model included age, sex, baPWV at baseline, marital status, educational levels, smoking status, drinking status, physical activity, sleep duration, lipid-lowering, antihypertensive medications, and history of coronary heart disease or stroke at baseline.

**Supplement Figure 2. The associations of mean arterial blood pressure with the risk of arterial stiffness.**


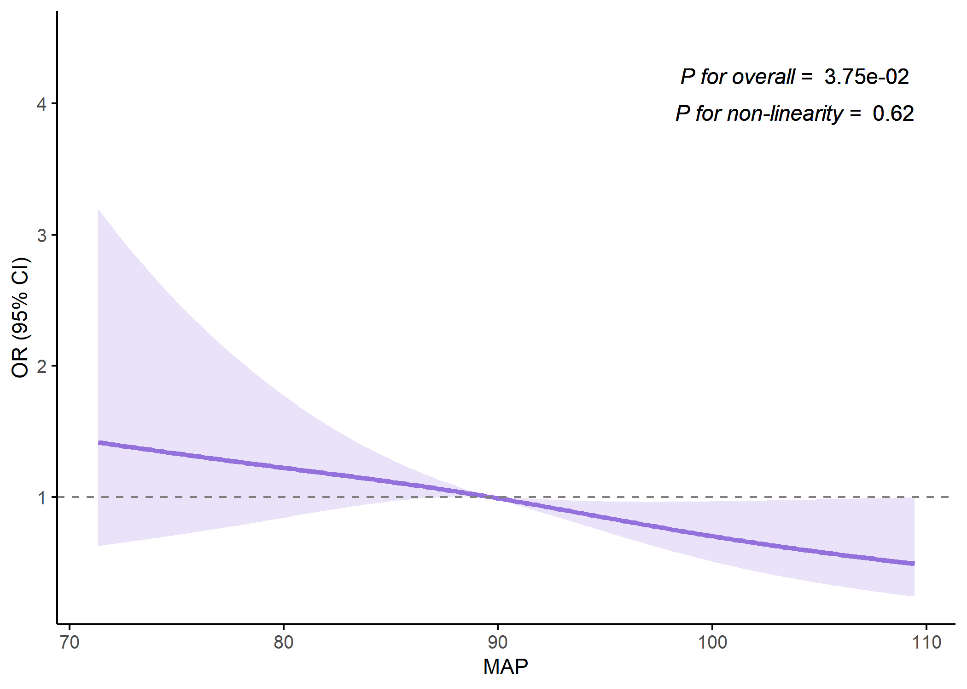


The data were fitted using restricted cubic spline logistic regression models with three knots placed at the 10th, 50th, and 90th percentiles of baseline MAP.

MAP: mean arterial blood pressure, CI: confidence interval.

Covariates in the model included age, sex, marital status, educational levels, smoking status, drinking status, physical activity, sleep duration, lipid-lowering, antihypertensive medications, and history of coronary heart disease or stroke at baseline.
